# Multi Vesicular Osseous Hydatid Disease of the Mandible- A Case Report

**Published:** 2010-03

**Authors:** H Shahoon, M Esmaeili, I Mobedi, M Nematollahi

**Affiliations:** 1 Department of Oral and Maxillofacial Surgery, Dental School of Shahed University, Tehran, Iran; 2 Department of Oral Medicine and Diagnostic Science, Dental School of Shahed University, Tehran, Iran; 3 Department of Medical Parasitology, School of Public Health, Tehran University of Medical Sciences, Tehran, Iran; 4 Private Practice, No.165, Mostofi Street, Yousef abad, Tehran, Iran

**Keywords:** *Echinococcus granulosus*, Hydatid disease, Mandible, Multi vesicular

## Abstract

Hydatid disease is a common and major public health issue caused by parasite *Echinococcus granulosus*. The highest prevalence of the parasite can be found in different parts of world like Africa, Australia, and South America. This infection can occurs in almost any part of the body. Here we present clinical, radiological, histological features and treatment of a multi vesicular osseous hydatid disease of the mandible in an Afghan 5 year old boy with a firm swelling in the right side of mandible.

## Introduction

Hydatid disease is a parasitic infection that has a worldwide geographic distribution and occurs in all continents including circumpolar, temperate, subtropical, and tropical zones (1, 2) and causes considerable health and economic problems (3). This disease is widely endemic in the South America, East Africa, and Mediterranean countries (4, 5). It is especially problematic in eastern and southeastern Turkey. In Iran, approximately 1% of all admissions to hospital surgical wards are associated to cystic echinococcosis, which is still considered endemic. During the last decade in Iran, there appears to have been a generally downward trend in the incidence of helminthiases (6).

The disease is caused by *Echinococcus granulosus*. The adult parasite (0.5–1cm) resides in the intestine of animals such as dogs, foxes, wolfs and jackals (7–9). Most hydatid cysts occur in the liver (59–75%) or in the lung (27%). Involvement of kidney (3%) or brain (1–2%) is rare. Spinal hydatid cysts account for 1% of all cases of hydatid disease (10–12).

Osseous hydatid disease is an infrequent entity that represent 0.5–2/5% of all hydatidosis (10). The vertebrae are the most commonly affected bones (50%), followed by the pelvis (25%) and long bones (15–25%) (13). Initial location of the lesions in long bones is metaphyseal or epiphyseal, later extending to the diaphysis (14). Involvement of jaw bones are very rare (10).

## Case Report

### Clinical Findings

**Fig. 1:**
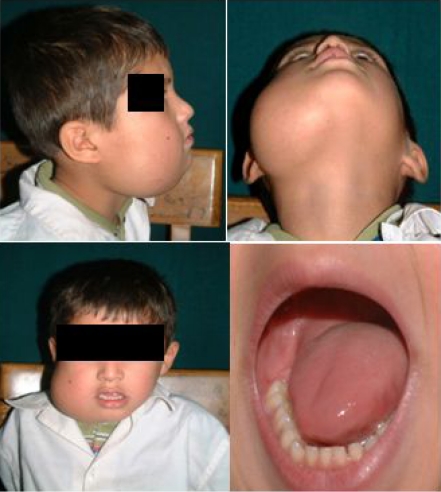
Firm swelling without pain measured 10×10 cm

An Afghan 5 year old boy with a firm swelling in the right side of mandible was referred to Taleqani University Hospital, Tehran, Iran. He lived in a village around Tehran and his father was a farmhand. His medical history was unremarkable. On examination, swelling was without pain (non-tender), firm & measured about 10×10 cm. The over lying skin of the swelling was normal. The dentition and oral mucosa were normal (Fig. 1). His general condition appeared to have little malnutrition.

### Radiographic findings

**Fig. 2:**
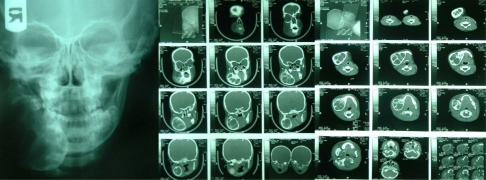
A multi lacunar lesion in the right side of mandible

In extra oral radiographic studies (OPG, CT Scan, PA of Mandible) a well-defined multi lacunar lesion in the right side of angle and body of the mandible was seen. According to the CT scan of the lesion, it seems that there were more than six lacunae in the cyst. No other abnormality findings were detected (Fig. 2).

### Laboratory tests

No abnormalities were found on standard blood tests. The patient's serum was assessed for hydatid cyst antibody by ELISA and the results were negative.

### Surgical findings

**Fig. 3:**
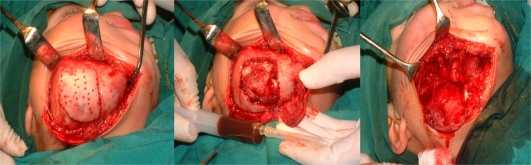
Multi lacunar cyst with thickened wall

On excisional biopsy, we observed multi lacunar cyst with well-defined, firm and thickened wall. The cyst was located without any attachment to the peripheral bone. Several septums, separated this cyst (Fig. 3).

**Fig. 4:**
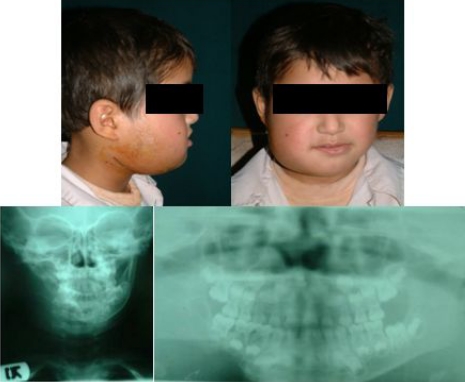
The patient's pictures after treatment

Hemovac tube was inserted to the site and soft tissues were sutured in three layers. Mebendazol (10 mg/ kg/day) was prescribed for 12 weeks. Patient visited a week after operation and was satisfied of the outcome. He was examined clinically and radiologically 18 months after surgery. No evidence of recurrence was seen and serologic test by ELIZA was negative (Fig. 4).

### Histological findings

**Fig. 5:**
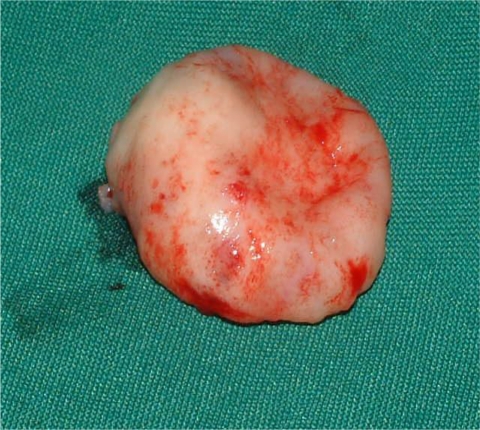
The full figure of the cyst

**Fig. 6:**
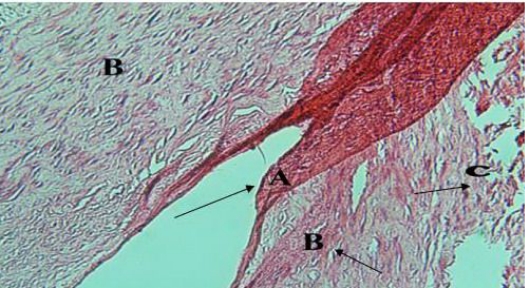
Histopathologic micrograph. Hematoxylin & eosin stain, original magnification×0.4 **A)** endo cyst (germinated layer). **B)** ecto cyst. **C)** peri

We excised the cyst and reshaped the body and angle of right side of mandible (Fig. 5). Then we rinsed the operation site with about 4 liters hypertonic saline. On histopathological examination, there was an osseous cyst, which seemed to be started in the bone marrow and had grown outwardly. The osseous cyst was multi vesicular and non-fertile. We could not find protoscolex in histopathological examination. The cyst wall was calcified and this means that the patient was in chronic phase of the disease. Peri cyst cells were completely absent and ectocyst tissue was caltected. Ectocyst laminated layers were suppressed by the infiltrating cells specially fibroblast in limited areas where overlapping laminated layers were detected, while endocyst “germinated layer” was found by seeing inside the cyst (Fig. 6).

## Discussion

Hydatid cysts can occur in any part of the body. Osseous hydatidosis is rare comparing to involvement of soft tissues. The prevalence of bone infection is 14% and 1.1% in endemic and non-endemic regions, respectively (15).

The diagnosis of hydatid cyst should be considered in patients with a cystic mass, who lived or visited in/an endemic area (16). Diagnosis of osseous hydatidosis is primarily based on the patient history, radiography such as X-ray and CT scans, seological tests such as ELIZA and histopathologic examination (17, 18). Radiographic appearance of hydatid cyst may include mono-locular, bi-locular, or as in this case multi-locular cyst. While early diagnosis is uncommon (17) serologic findings may help the diagnosis, but they are not reliable alone especially in the osseous hydatidosis cases, which the serologic tests usually are negative as it is in this case (19). Immunologic diagnostic procedures for serum antibody detection such as IgG ELISA, Indirect Hemagglotination Antibody Test (IHAT), Latex Agglutination Test (LAT), Immuno–Fluorescence Antibody Test (IFAT), Immune Electro Phoresis (IEP) and some other tests are used for etiological confirmation of imaging structures suggestive of cystic echinococcosis or for diagnosis in cases of uncharacteristic imaging findings (20).

Osseous hydatidosis may manifest as bone pain and deformity, particularly among patients in 30–60 years old ages (21). Hydatid cysts may lie dormant in the bone; therefore mostly appear in adulthood. Potential complications include pathologic fracture, infection, and fistulization of the abscess (22). Men more than women are at infection risk (1) and the peak age of disease in Iran is 21–40 years (9, 15). Hydatid bone disease is often asymptomatic and is therefore usually diagnosed at an advanced stage when the lesions have become extensive (18). In the present case, the only clinical manifestation of the disease was bone deformity. It seems that the lesion in this infected Afghan boy had started the growth in the bone marrow of the mandibular bone and continued its growth outwardly and expanded the bone but did not perforate the cortex of the mandible as is obvious in the CT scans of the lesion. The cyst was calcified in some parts and was developed into a large size in the 5 years old boy. We think that the equilibrium between the pathogenicity of the *E. granulosus* and host defense had dictated the progress and structure of the cyst and maybe calcification in this case had been a protective mechanism to decrease the growth rate of the lesion.

The differential diagnosis of skeletal hydatid cyst includes other infections disease (e.g. tuberculosis), fibroma, lymphoma, giant cell tumors, brown tumors, metastatic lesions, and other benign cystic lesions (16). The presence of a periosteal reaction, osteosclerosis and calcification are not specific for hydatid bone disease (18).

Curettage of the lesion and drug therapy (Albendazol or mebendazol) have been reported to be sufficient treatment (23) but many authors have advocated wide resection of the involved bone, along with the surrounding soft tissue as the only definitive treatment of the condition (24, 25).
